# Development and validation of a nomogram to predict leptomeningeal metastases in lung adenocarcinoma: Cervical lymph node metastasis is an important association factor

**DOI:** 10.1002/cam4.7206

**Published:** 2024-04-30

**Authors:** Xiaoyu Hua, Weifeng Feng, Minting Ye, Mingyao Lai, Xiaojun Yu, Mengnan Sun, Juan Li, Ruyu Ai, Yanlin He, Linbo Cai, Changzheng Shi, Xiangning Liu

**Affiliations:** ^1^ Department of Medical Imaging Centre The First Affiliated Hospital, Jinan University Guangzhou China; ^2^ The First Affiliated Hospital, Jinan University Guangzhou China; ^3^ Department of Medical Oncology Guangdong Sanjiu Brain Hospital Guangzhou China; ^4^ Department of Medical Imaging Centre Inner Mongolia People's Hospital Hohhot China; ^5^ Clinical Research Platform for Interdiscipline of Stomatology The First Affiliated Hospital of Jinan University Guangzhou China; ^6^ Department of Stomatology College of Stomatology, Jinan University Guangzhou China

**Keywords:** leptomeningeal metastases, lung adenocarcinoma, nomogram, prediction, staging

## Abstract

**Background:**

The goal of this study was to create a nomogram using routine parameters to predict leptomeningeal metastases (LMs) in advanced lung adenocarcinoma (LAC) patients to prevent needless exams or lumbar punctures and to assist in accurately diagnosing LMs.

**Methods:**

Two hundred and seventy‐three patients with LMs and brain metastases were retrospectively reviewed and divided into derivation (*n* = 191) and validation (*n* = 82) cohorts using a 3:7 random allocation. All LAC patients with LMs had positive cerebrospinal fluid cytology results and brain metastases confirmed by magnetic resonance imaging. Binary logistic regression with backward stepwise selection was used to identify significant characteristics. A predictive nomogram based on the logistic model was assessed through receiver operating characteristic curves. The validation cohort and Hosmer–Lemeshow test were used for internal validation of the nomogram.

**Results:**

Five clinicopathological parameters, namely, gene mutations, surgery at the primary lung cancer site, clinical symptoms of the head, N stage, and therapeutic strategy, were used as predictors of LMs. The area under the curve was 0.946 (95% CI 0.912–0.979) for the training cohort and 0.861 (95% CI 0.761–0.961) for the internal validation cohort. There was no significant difference in performance between the two cohorts (*p* = 0.116). In the internal validation, calibration plots revealed that the nomogram predictions were well suited to the actual outcomes.

**Conclusions:**

We created a user‐friendly nomogram to predict LMs in advanced lung cancer patients, which could help guide treatment decisions and reduce unnecessary lumbar punctures.

## 1 BACKGROUND

Leptomeningeal metastasis (LM) is a distressing and catastrophic occurrence in advanced cancer patients. LM involves the dissemination of cancerous cells to the leptomeninges, the subarachnoid space, and additional compartments within the cerebrospinal fluid (CSF) system.^1^ Approximately 3–5% of individuals diagnosed with advanced non‐small cell lung cancer are affected by LMs. However, due to improved results from new molecular therapeutics, their incidence has increased within specific patient subgroups harboring targetable mutations.^2^, ^3^ Without tumor‐specific therapy, patients with LM have a survival time of approximately 1–3 months, which can be increased to 3–11 months with new therapies.^4^, ^5^, ^6^ These findings suggest that early diagnosis and reasonable treatment, as opposed to treating LM as a terminal illness, could increase patient survival.

Leptomeningeal metastasis is challenging to diagnose and is based on clinical, CSF, and neuroimaging manifestations. The gold standard for the diagnosis of LM remains positive CSF cytology, although recurrent CSF collection through intrathecal puncture is frequently needed. According to some reports, the sensitivity of the first intrathecal puncture is as low as 50%; however, with a second CSF analysis, the sensitivity can increase to 75%–85%.^7^ At least 10 mL of CSF must be collected, and the analysis must be completed swiftly to increase sensitivity.^8^ A meningeal biopsy is rarely required for a diagnosis. The involvement of many central nervous system areas results in pleomorphic clinical presentations in LM patients. Weakness, headache or back pain, nausea, vomiting, cranial nerve paralysis (such as diplopia or visual disruption), hearing loss, changes in mental state, difficulty walking, seizures, and focal neurological abnormalities are common symptoms.^9^ The majority of symptoms and indications are vague and easy to ignore. The sensitivity of gadolinium‐enhanced magnetic resonance imaging (MRI) for detecting LMs in solid tumors typically ranges from 70% to 85%, while its specificity ranges from 75% to 90%.^2^, ^10^ Due to the low sensitivity of CSF cytology and MRI and the presence of atypical clinical symptoms, patients at risk of LM may experience delays or mistaken diagnoses.

Diagnostic prediction models are tools that incorporate multiple predictors by assigning relative weights to each predictor. Through this process, these models generate risk or probability estimates, which are used to evaluate the likelihood of a particular disease or condition. These models aid in determining whether additional testing or immediate treatment initiation is warranted, offering guidance to patients.^11^, ^12^ To date, several models to predict LMs have been published^13^, ^14^, ^15^; however, this study is the first to classify N3 metastasis into hilar and mediastinal, subclavian, and cervical lymph nodes and to determine the effect of treatment methods before LM. The objective of the present study was to develop a nomogram utilizing routine clinicopathological parameters to predict LM. Furthermore, the utilization of this nomogram is specifically restricted to patients diagnosed with lung adenocarcinoma (LAC). This decision is based on evidence from several studies indicating that the adenocarcinoma subtype of LAC exhibits a more favorable response to radiation therapy and targeted therapeutic agents, such as EGFR inhibitors.^16^, ^17^ By focusing on LAC patients, we aimed to develop a more specialized and tailored predictive tool that can accurately assess the risk of LM in this particular subgroup. This limitation ensures that the nomogram's predictions align closely with the characteristics and treatment responses observed in patients with LAC, facilitating more informed clinical decision‐making for this specific population.

## 2 MATERIALS AND METHODS

### 2.1 Patient selection and data processing

**FIGURE 1:**
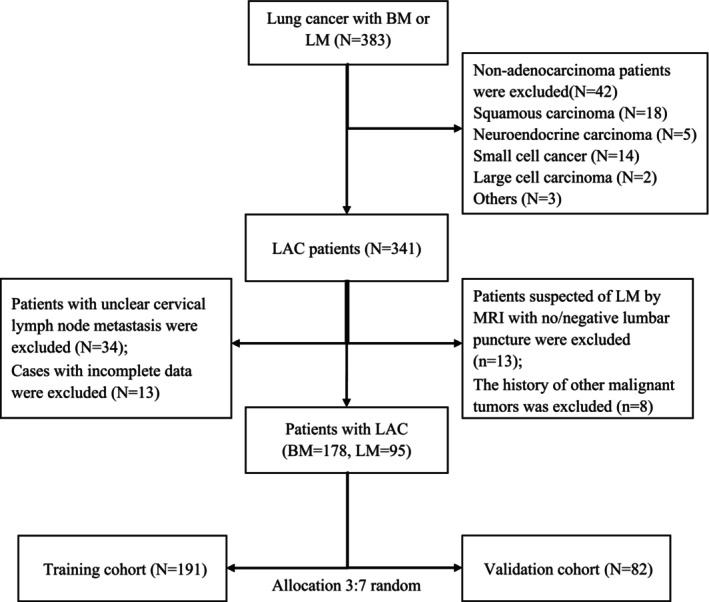
Study flowchart. BM, brain metastases; LAC, lung adenocarcinoma cell; LM, leptomeningeal metastasis; MRI, magnetic resonance imaging.

In alignment with the principles outlined in the Declaration of Helsinki, a retrospective observational study was carried out at the Guangdong Sanjiu Brain Hospital. The study protocol was approved by the Ethics Committee of Guangdong Sanjiu Brain Hospital. In accordance with their guidelines, it was determined that the study was exempt from the requirement for informed consent. The electronic files in the Guangdong Sanjiu Brain Hospital's Hospital Information System (HIS) were used to screen patients from January 2014 to November 2022. Data were obtained from 383 consecutive patients with brain metastasis (BM) and LM of lung cancer. Forty‐two patients with non‐LAC were excluded. A total of 68 patients were disqualified for the following reasons: (i) incomplete clinical information, missing at least two of the following terms: gene mutations, surgery on the primary site of lung cancer, clinical symptoms of the head, tumor‐node‐metastasis (TNM) staging, and therapy strategy; (ii) a history of other malignancies; (iii) ambiguous cervical lymph node metastases; and (iv) suspected LMs on MRI with no lumbar puncture or negative lumbar puncture. The study cohort of 273 patients was then split at random into training (70%, *n* = 191) and validation (30%, *n* = 82) sets. Figure 1 presents the study flowchart. In this study, MRI screening was used to identify BM. Positive CSF cytology results were required for the diagnosis of LM.

### 2.2 Data collection

Each patient's baseline clinical and pathological characteristics were acquired from their electronic medical records. These characteristics encompassed a range of information, including age, sex, smoking status, location of the primary tumor, clinical symptoms, family history, genetic mutations, marital status, primary site surgery, past medical history, extracranial metastases, and TNM staging (initial diagnosis of LAC). Treatment before LM included no therapy, systemic therapy, local therapy, or local and systemic therapy. Systemic therapy included chemotherapy, targeted therapy, and immunotherapy. Local treatment included surgical resection of BMs and brain radiotherapy.

### 2.3 Development and validation of the nomogram

For variables with a low proportion of missing data, missing values were imputed. Clinical reasoning and statistical methods were used to categorize continuous variables. A cutoff of 60 years was used to divide patients into two categories by age. To identify significant characteristics, we utilized binary logistic regression in combination with the backward stepwise selection method. The performance of the nomogram in predicting outcomes was evaluated using two metrics: the Brier score and the receiver operating characteristic (ROC) curve. The Hosmer–Lemeshow test and validation cohort were used for internal validation of the nomogram. Additionally, we employed decision curve analysis (DCA) to examine the clinical applicability of the nomogram by plotting the net benefit (NB) across a range of risk thresholds that align with practical clinical decision‐making.

### 2.4 Statistical analysis

Statistical analysis was performed using R Statistical Software version 4.1.2 and IBM SPSS Statistics version 27.0. Proportions are used to summarize categorical variables, providing a clear understanding of the distribution within each category. The chi‐square test was used to compare categorical variables. We utilized various R packages, including regplot, rms, rmda, survival, and pROC, to create informative graphical representations for our analysis. These packages enabled us to generate essential graphs such as the nomogram, ROC curve, DCA, and calibration plot. All *p* values were two‐sided, with *p* < 0.05 indicating statistical significance and 95% confidence intervals (CIs).

## 3 RESULTS

### 3.1 Clinicopathological characteristics

A total of 383 patients were evaluated, 273 of whom were eventually included in the nomogram. Figure 1 depicts the study flow chart. Table 1 displays the baseline characteristics of the study population. The majority of patients in the cohort were less than 60 years old (59.3%), were male (59.7%), were nonsmokers (71.8%), did not have surgery at the primary site (87.9%), had no family history (90.8%), were married (94.5%), and had no past medical history (71.8%). The most prevalent driver mutation was an EGFR mutation (51.3%), while the most common clinical symptoms were headache and dizziness (53.8%). The brain (91.6%) and lymph nodes (90.1%) were the most prevalent sites of tumor metastasis among these individuals, followed by bone (52.7%) and lung (24.9%). The majority of patients did not have pleural (82.8%), hepatorenal (85.3%), or adrenal metastases (85.0%). The most common N stage was N2 (40.7%), and the most common therapeutic modality was local and systemic therapy (58.2%).

**TABLE 1:** Baseline characteristics.

Characteristics	All patient (*n* = 274)	BM (*n* = 178)	LM (*n* = 96)	*p* Value
Age, *n* (%)				
<60	162 (59.3)	91 (51.1)	71 (74.7)	<0.001
≥60	111 (40.7)	87 (48.9)	24 (25.3)	
Sex, *n* (%)				
Male	163 (59.7)	108 (60.7)	55 (57.9)	0.656
Female	110 (40.3)	70 (39.3)	40 (42.1)	
Gene mutations, *n* (%)				
Wild type	60 (22.0)	49 (27.5)	11 (11.6)	0.001
ALK	35 (12.8)	23 (12.9)	12 (12.6)	
EGFR	140 (51.3)	76 (42.7)	64 (67.4)	
KRAS	10 (3.7)	9 (5.1)	1 (1.1)	
Others	28 (10.2)	21 (11.8)	7 (7.3)	
Smoking, *n* (%)				
Yes	77 (28.2)	54 (30.3)	23 (24.2)	0.284
No	196 (71.8)	124 (69.7)	72 (75.8)	
Primary site surgery, *n* (%)				
Yes	33 (12.1)	18 (10.1)	15 (15.8)	0.017
No	240 (87.9)	160 (89.9)	80 (84.2)	
Fhistory, *n* (%)				
Yes	25 (9.2)	10 (5.6)	15 (15.8)	0.006
No	248 (90.8)	168 (94.4)	80 (84.2)	
Marital, *n* (%)				
Single	13 (4.8)	8 (4.5)	5 (5.3)	0.564
Married	258 (94.5)	168 (94.4)	90 (94.7)	
DSW	2 (0.7)	2 (1.1)	0 (0.0)	
Phistory, *n* (%)				
Yes	77 (28.2)	52 (29.2)	25 (26.3)	0.612
No	196 (71.8)	126 (70.8)	70 (73.7)	
Symptom, *n* (%)				
No	60 (22.0)	53 (29.8)	7 (7.4)	<0.001
Headache, Dizziness	147 (53.8)	75 (42.1)	72 (75.8)	
Distal limb weakness, ataxia, stoit	42 (15.4)	39 (21.9)	3 (3.2)	
Apsychia	2 (0.7)	1 (0.6)	1 (1.1)	
Epilepsy	6 (2.2)	2 (1.1)	4 (4.2)	
Cranial nerve paralysis	16 (5.9)	8 (4.5)	8 (8.3)	
Lung metastasis, *n* (%)				
Yes	68 (24.9)	39 (14.3)	29 (30.5)	0.117
No	205 (75.1)	139 (78.1)	66 (69.5)	
Pleural metastasis, *n* (%)				
Yes	47 (17.2)	23 (12.9)	24 (25.3)	0.01
No	226 (82.8)	155 (87.1)	71 (74.7)	
Brain metastases, *n* (%)				
Yes	250 (91.6)	178 (100.0)	72 (75.8)	0.998
No	23 (8.4)	0 (0.0)	23 (24.2)	
Osseous metastasis, *n* (%)				
Yes	144 (52.7)	81 (45.5)	63 (66.3)	0.001
No	129 (47.3)	97 (54.5)	32 (33.7)	
Adrenal gland metastasis, *n* (%)				
Yes	41 (15.0)	27 (15.2)	14 (14.7)	0.924
No	232 (85.0)	151 (84.8)	81 (85.3)	
Hepatorenal metastasis, *n* (%)				
Yes	40 (14.7)	23 (12.9)	17 (17.9)	0.268
No	233 (85.3)	155 (87.1)	78 (82.1)	
Others metastasis, *n* (%)				
Yes	13 (4.8)	8 (4.5)	5 (5.3)	0.776
No	260 (95.2)	170 (95.5)	90 (94.7)	
T staging, *n* (%)				
T1	42 (15.3)	29 (16.3)	13 (13.6)	0.171
T2	111 (40.7)	76 (42.7)	35 (36.8)	
T3	25 (9.2)	19 (10.7)	6 (6.3)	
T4	95 (34.8)	54 (30.3)	41 (43.3)	
N staging, *n* (%)				
N0	27 (9.9)	19 (10.7)	8 (8.3)	<0.001
N1	33 (12.0)	20 (11.2)	13 (13.7)	
N2	111 (40.7)	87 (48.9)	24 (25.3)	
N3 (Lung and mediastinal)	28 (10.3)	19 (10.7)	9 (9.5)	
N3 (Supraclavicular)	55 (20.1)	29 (16.3)	26 (27.4)	
N3 (cervical lymph nodes)	19 (7.0)	4 (2.2)	15 (15.8)	
Therapeutic method, *n* (%)				
No therapy	15 (5.5)	2 (1.1)	13 (13.7)	<0.001
Systemic therapy	77 (28.2)	19 (10.7)	58 (61.0)	
Local therapy	22 (8.1)	21 (11.8)	1 (1.1)	
Local and systemic therapy	159 (58.2)	136 (76.4)	23 (24.2)	

Abbreviations: BM, brain metastases; DSW, divorced & separated &widowed; Fhistory, family history; LM, leptomeningeal metastasis; Phistory, past medical history.

### 3.2 Nomogram development

Univariable analysis revealed that age (*p* < 0.001), gene mutations (*p* = 0.002), primary site surgery (*p* = 0.002), family history (*p* = 0.008), clinical symptoms of the head (*p* < 0.001), pleural metastasis (*p* = 0.011), osseous metastasis (*p* < 0.001), N stage (*p* = 0.001), and therapeutic method (*p* < 0.001) were associated with LM. According to the multivariable logistic regression analysis, gene mutations (*p* = 0.007; ALK: OR = 3.275, 95% CI 0.672–15.953, *p* = 0.142; EGFR: OR = 5.80, 95% CI 1.699–19.929, *p* = 0.005; KRAS: OR = 0.325, 95% CI 0.014–7.479, *p* = 0.482; others: OR = 0.910, 95% CI 0.171–4.856, *p* = 0.912), primary site surgery (OR = 7.742, 95% CI 1.825–32.846, *p* = 0.006), clinical symptoms of the head (*p* < 0.001; headache, dizziness: OR = 18.695, 95% CI 4.335–80.628, *p* < 0.001; distal limb weakness, ataxia, stoit: OR = 0.885, 95% CI 0.107–7.292, *p* = 0.911; apsychia: OR = 18.819, 95% CI 0.003–123.19, *p* = 0.513; epilepsy: OR = 1.944, 95% CI 0.123–30.760, *p* = 0.637; cranial nerve paralysis: OR = 18.127, 95% CI 2.299–142.951, *p* = 0.006), N staging (*p* = 0.001; N1: OR = 1.384, 95% CI 0.232–8.243, *p* = 0.721; N2: OR = 0.808, 95% CI 0.177–3.688, *p* = 0.783; N3 lung and mediastinal: OR = 2.754, 95% CI 0.434–17.485, *p* = 0.283; N3 supraclavicular: OR = 4.069, 95% CI 0.801–20.673, *p* = 0.091; N3 cervical lymph nodes: OR = 23.321, 95% CI 3.067–177.331, *p* = 0.002), and therapeutic method (*p* < 0.001; systemic therapy: OR = 0.187, 95% CI 0.030–1.184, *p* = 0.075; local therapy: OR = 0.006, 95% CI 0.001–0.092, *p* < 0.001; local and systemic therapy: OR = 0.006, 95% CI 0.001–0.040, *p* < 0.001) were significantly associated with LM (Table 2). N3 (cervical lymph nodes) had the strongest positive association on LM.

**TABLE 2:** Univariate and multivariate analyses of the prognostic factors associated with LM in the entire cohort (*N* = 273).

Characteristics	Univariable OR (95% CI)	*p* Value	Multivariable OR (95% CI)	*p* Value
Age (<60 vs. ≥60)	0.354 (0.204–0.612)	<0.001	0.384 (0.138–1.070)	0.067
Sex (male vs. female)	1.122 (0.676–1.862)	0.655		
Gene mutations		0.002		0.007
Wild type	(reference)		(reference)	
ALK	2.324 (0.893–6.048)	0.841	3.275 (0.672–15.953)	0.142
EGFR	3.751 (1.801–7.812)	<0.001	5.800 (1.699–19.929)	0.005
KRAS	0.495 (0.057–4.322)	0.525	0.325 (0.014–7.479)	0.482
Others	1.485 (0.506–4.358)	0.472	0.910 (0.171–4.856)	0.912
Smoking (yes vs. no)	0.734 (0.416–1.294)	0.285		
Primary site surgery (yes vs. no)	23.886 (3.238–176.205)	0.002	7.742 (1.825–32.846)	0.006
Fhistory (yes vs. no)	3.150 (1.355–7.321)	0.008	4.561 (0.897–23.188)	0.067
Phistory (yes vs. no)	0.865 (0.495–1.514)	0.612		
Clinical symptoms of the head		<0.001		<0.001
No	(reference)		(reference)	
Headache, dizziness	7.269 (3.101–17.039)	<0.001	18.695 (4.335–80.628)	<0.001
Distal limb weakness, ataxia, stoit	0.582 (0.142–2.396)	0.454	0.885 (0.107–7.292)	0.911
Apsychia	7.571 (0.424–135.109)	0.169	18.819 (0.003–123.19)	0.513
Epilepsy	15.143 (2.330–98.395)	0.004	1.944 (0.123–30.760)	0.637
Cranial nerve paralysis	6.625 (1.834–23.938)	0.004	18.127 (2.299–142.951)	0.006
Lung metastasis (yes vs. no)	1.566 (0.892–2.749)	0.118		
Pleural metastasis (yes vs. no)	2.278 (1.205–4.308)	0.011	2.965 (0.962–9.136)	0.058
Brain metastases (yes vs. no)	0.001 (0.001–1.943)	0.998		
Osseous metastasis (yes vs. no)	2.358 (1.405–3.957)	<0.001	1.726 (0.748–3.981)	0.201
Adrenal gland metastasis (yes vs. no)	0.967 (0.480–1.946)	0.924		
Hepatorenal metastasis (yes vs. no)	1.469 (0.742–2.909)	0.271		
Others metastasis (yes vs. no)	1.181 (0.375–3.714)	0.777		
T staging		0.176		
T1	(reference)			
T2	1.027 (0.477–2.212)	0.945		
T3	0.704 (0.228–2.174)	0.542		
T4	1.694 (0.784–3.658)	0.181		
N staging		0.001		0.001
N0	(reference)		(reference)	
N1	1.544 (0.523–4.553)	0.431	1.384 (0.232–8.243)	0.721
N2	0.655 (0.256–1.680)	0.379	0.808 (0.177–3.688)	0.783
N3 (Lung and mediastinal)	1.125 (0.358–3.536)	0.841	2.754 (0.434–17.485)	0.283
N3 (Supraclavicular)	2.129 (0.798–5.680)	0.131	4.069 (0.801–20.673)	0.091
N3 (cervical lymph nodes)	8.906 (2.245–35.330)	0.002	23.321 (3.067–177.331)	0.002
Therapeutic method		<0.001		<0.001
No therapy	(reference)		(reference)	
Systemic therapy	0.470 (0.097–2.272)	0.347	0.187 (0.030–1.184)	0.075
Local therapy	0.007 (0.001–0.089)	<0.001	0.006 (0.001–0.092)	<0.001
Local and systemic therapy	0.026 (0.006–0.123)	<0.001	0.006 (0.001–0.040)	<0.001

*Note*: Systemic therapy included chemotherapy, targeted therapy, and immunotherapy. Local treatment includes surgical resection of brain metastases and brain radiotherapy.

Abbreviations: Fhistory, family history; Phistory, past medical history.

**FIGURE 2:**
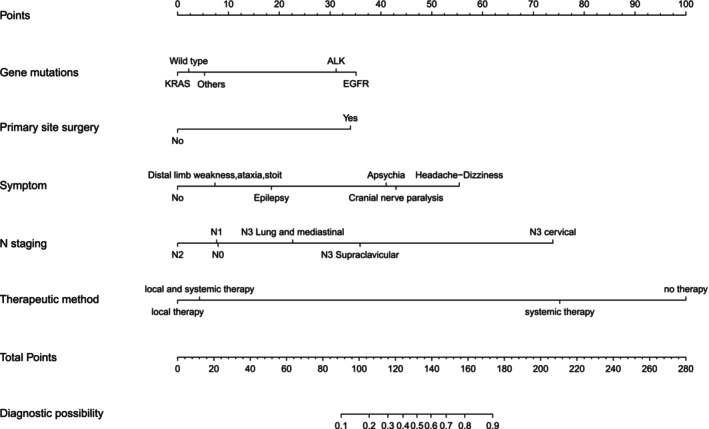
Nomogram for predicting leptomeningeal metastasis in advanced lung adenocarcinoma patients.

There was no substantial collinearity among the continuous variables. The construction of the nomogram involved selecting variables that demonstrated statistical significance in the multivariable analysis. This selection process was performed using the backward stepwise selection method. The total score for each patient was determined by summing the scores of each characteristic to estimate the likelihood of developing LM (Figure 2).

### 3.3 Model comparison and validation

**FIGURE 3:**
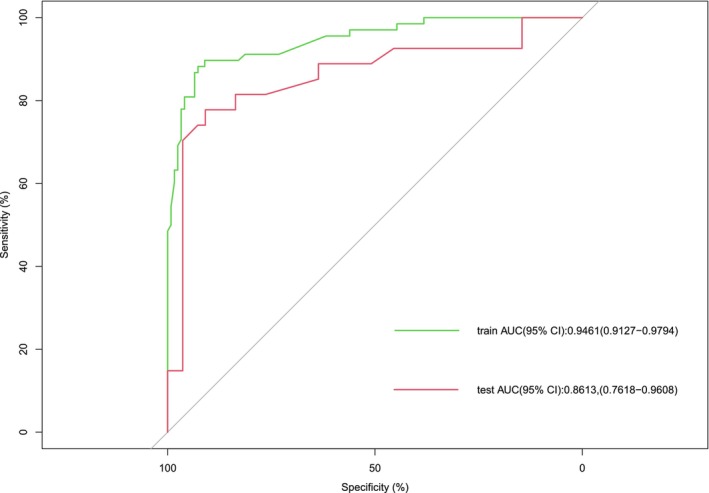
Receiver operating characteristic curves were plotted to assess the discriminatory power of the training and testing cohorts.

**FIGURE 4:**
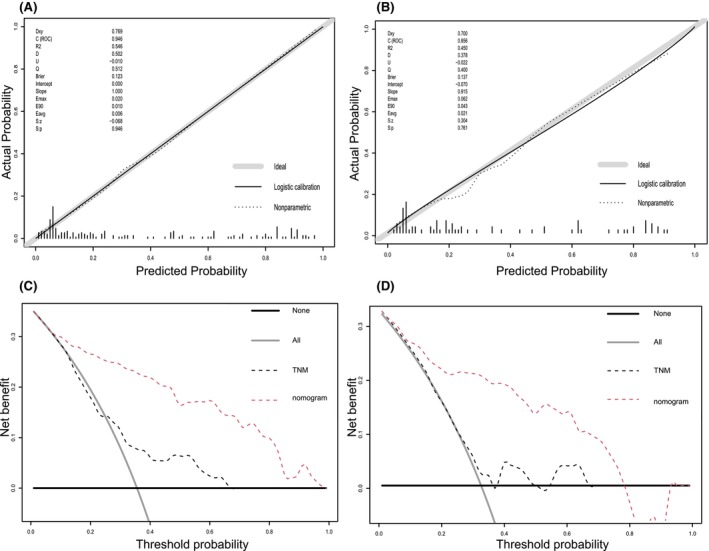
Calibration plot of the nomogram and decision curve analysis (DCA). Validity of the predictive performance of the nomogram in the training cohort (A) and validation cohort (B). DCA of the nomogram in the training cohort (C) and validation cohort (D). The decision curve showed that the nomogram had a greater net benefit than did the TNM system.

Our classifier based on five clinicopathological variables worked well in the training group, with an area under the curve (AUC) of 0.946 (95% CI 0.912–0.979) (Figure 3). Figure 4A displays the internal bootstrapped calibration plot, which assessed the agreement between the nomogram's predictions regarding the likelihood of LM and the actual observations. In the testing set, the nomogram showed comparable AUC values of 0.861 (95% CI 0.761–0.961) for the estimation of LM (Figure 3). There was also a good calibration curve for LM estimation (Figure 4B). The Hosmer–Lemeshow test demonstrated that the nomogram was well fitted, with *p* values of 0.972 and 0.072, respectively.

Additionally, in the DCA, the nomogram outperformed the TNM system in predicting LM when the threshold probability exceeded 0.1. This analysis is depicted in Figure 4C,D, illustrating that the nomogram yielded a greater NB in LM prediction than did the TNM system. This finding indicates that utilizing the nomogram in clinical decision‐making resulted in better outcomes and increased the overall efficacy of predicting LM occurrence.

## 4 DISCUSSION

In this study, we developed an easy‐to‐use nomogram that incorporated five commonly used clinical and pathological predictors, namely, gene mutations, surgery at the primary site of lung cancer, clinical symptoms of the head, N stage, and therapeutic strategy. The nomogram exhibited excellent predictive performance in terms of discrimination and calibration. Our nomogram exhibited results consistent with those of previously published models, further validating its usefulness in clinical practice.

The frequency of LM has increased in recent decades as patient survival has increased via improved tolerability and highly successful therapeutic alternatives, and LM has emerged as a major contributor to morbidity and mortality rates.^18^, ^19^ Diagnosing LM can be challenging and relies on clinical evaluation, CSF analysis, and radiographic findings. The gold standard for LM diagnosis remains a positive CSF cytology test. However, the positive rate of the first CSF puncture is low. To enhance sensitivity, repeated CSF punctures are often necessary, typically ensuring the collection of at least 10 mL of CSF, and the analysis must be performed quickly, which may increase patient susceptibility to infections, hemorrhage, or brain herniation.^20^, ^21^ In the early stages of LM, neuroimaging may show a false negative finding or aberrant enhancement in situations such as inflammation, low intracranial pressure, and recent radiation therapy or surgical procedures comparable to those of LM. In addition, some people cannot undergo MRI because of contraindications, such as internal metal implants and claustrophobia.^22^, ^23^, ^24^ In comparison to conventional tools, CSF biopsy has become a topic of interest in research in recent years; it could be a useful tool for diagnosing and monitoring LM and understanding its specific biology, as it allows for the identification of mechanisms that contribute to treatment resistance, such as the CellSearch technique and the evaluation of CSF‐circulating tumor DNA.^25^, ^26^, ^27^ Nonetheless, corresponding guidelines are unavailable, and the relatively high cost of this technology restricts its clinical applicability. To address these challenges, a more accurate and cost‐effective diagnostic model should be developed.^20^

To date, there are several predictive models for pial metastasis based on clinical parameters.^13^, ^14^, ^15^, ^28^ Our nomogram's discriminative capacity is comparable to that of models created by Tianqi Gao et al.^13^ We used three of the same predictors, namely, genetic mutations, clinical symptoms in the head, and stage N disease. In addition, we added a previously unreported relationship between primary lung cancer surgery, treatment strategy, and LM. Our study provides, for the first time, evidence that local treatment, including surgical resection of BMs and brain radiotherapy, is a significant protective factor against the development of LM. Moreover, for the first time, we refined N3 to include hilar and mediastinal lymph node, subclavian lymph node, and deep cervical lymph node metastases.^15^, ^29^ In addition, we found that deep cervical lymph node metastasis is an influential association factor for LM in lung cancer patients. Recent studies have shown that the meningeal lymphatic vessels are directly connected to the lymphatic vessels of the deep cervical lymph nodes that pass through the lamina cribrosa into the nasal mucosa.^30^, ^31^ More research is needed to determine whether cervical metastatic lymph nodes reflux into the meninx via lymphatic vessels and induce LM.

In clinical practice, when a lung cancer patient develops emerging clinical symptoms in the head, the clinician will play a critical role in deciding whether to perform MRI or lumbar puncture. Clinicians were unable to determine whether the head symptoms were caused by BM or meningeal metastasis in patients with MRI‐negative meningeal metastases, as most lung cancer patients also have concurrent BM disease. At this point, a lumbar puncture is needed. However, a lumbar puncture requires rigorous techniques and sampling requirements, resulting in a low positive rate. Using our predictive model to select suitable high‐risk patients for lumbar puncture not only improved the positive rate but also avoided unnecessary punctures.

However, several limitations should be noted. First, our models have not undergone external validation, suggesting that their predictive performance may be exaggerated. Before conversion to clinical practice, external validation of the model is needed. Second, in our study, we only included patients with confirmed LM who had positive CSF cytology. This may limit the applicability of our study to patients with probable LM who had only neurological symptoms and imaging manifestations. Third, our study had a constrained sample size, resulting in the possibility of selection bias, similar to previous retrospective studies.

## 5 CONCLUSIONS

In the current investigation, we used five normal clinical items to create a nomogram that predicted LM. The nomogram, which has strong discrimination and calibration, could assist oncologists in making a diagnosis and treatment plan, eliminating unnecessary lumbar punctures, and improving the diagnostic confidence of radiologists and pathologists.

## AUTHOR CONTRIBUTIONS

**Xiaoyu Hua:** Data curation (equal); project administration (equal); writing – original draft (equal). **Weifeng Feng:** Data curation (equal); project administration (equal); writing – original draft (equal). **Minting Ye:** Data curation (equal); project administration (equal); writing – original draft (equal). **Mingyao Lai:** Data curation (equal); project administration (equal); writing – original draft (equal). **Xiaojun Yu:** Formal analysis (equal); investigation (equal); software (equal). **Mengnan Sun:** Data curation (equal); investigation (equal). **Juan Li:** Data curation (equal); validation (equal). **Ruyu Ai:** Data curation (equal); investigation (equal). **Yanlin He:** Validation (equal); visualization (equal). **Linbo Cai:** Conceptualization (equal); methodology (equal); supervision (equal); writing – review and editing (equal). **Changzheng Shi:** Conceptualization (equal); methodology (equal); supervision (equal); writing – review and editing (equal). **Xiangning Liu:** Conceptualization (equal); methodology (equal); supervision (equal); writing – review and editing (equal).

## FUNDING INFORMATION

This study was supported by the Guangzhou Municipal Science and Technology Project, China (202002030086), the Science and Technology Projects in Guangzhou (2023A03J1030), and the Clinical Frontier Technology Program of the First Affiliated Hospital of Jinan University, China (JNU1AF‐CFTP‐2022‐a01210).

## CONFLICT OF INTEREST STATEMENT

The authors declare that they have no known competing financial interests or personal relationships that could have appeared to influence the work reported in this paper.

## ETHICS STATEMENT

This retrospective study was approved by the Ethics Committee of the Guangdong Sanjiu Brain Hospital (No. 2020‐010‐089), and there was informed consent exemption for all patients.

## Data Availability

The datasets used and/or analyzed during the current study are available from the corresponding author on reasonable request.
